# Evaluating ELISA, Immunofluorescence, and Lateral Flow Assay for SARS-CoV-2 Serologic Assays

**DOI:** 10.3389/fmicb.2020.597529

**Published:** 2020-12-11

**Authors:** Moïse Michel, Amar Bouam, Sophie Edouard, Florence Fenollar, Fabrizio Di Pinto, Jean-Louis Mège, Michel Drancourt, Joana Vitte

**Affiliations:** ^1^Aix-Marseille Univ, IRD, APHM, MEPHI, Marseille, France; ^2^IHU Méditerranée Infection, Marseille, France; ^3^Aix-Marseille Univ, IRD, APHM, VITROME, Marseille, France

**Keywords:** COVID-19 – diagnosis – ELISA – human – IgG antibodies – SARS-CoV-2 – standardization, IgG serology, IgM serology, lateral flow assay, ELISA, indirect immunofluorescence

## Abstract

**Background:**

The SARS-CoV-2 outbreak has emerged at the end of 2019. Aside from the detection of viral genome with specific RT-PCR, there is a growing need for reliable determination of the serological status. We aimed at evaluating five SARS-CoV-2 serology assays.

**Methods:**

An in-house immunofluorescence assay (IFA), two ELISA kits (EUROIMMUN^®^ ELISA SARS-CoV-2 IgG and NovaLisa^®^ SARS-CoV-2 IgG and IgM) and two lateral flow assays (T-Tek^®^ SARS-CoV-2 IgG/IgM Antibody Test Kit and Sure Bio-tech^®^ SARS-CoV-2 IgM/IgG Antibody Rapid Test) were compared on 40 serums from RT-PCR-confirmed SARS-CoV-2 infected patients and 10 SARS-CoV-2 RT-PCR negative subjects as controls.

**Results:**

Control subjects tested negative for SARS-CoV-2 antibodies with all five systems. Estimated sensitivities varied from 35.5 to 71.0% for IgG detection and from 19.4 to 64.5% for IgM detection. For IgG, in-house IFA, EuroImmun, T-Tek and NovaLisa displayed 50–72.5% agreement with other systems except IFA vs EuroImmun and T-Tek vs NovaLisa. Intermethod agreement for IgM determination was between 30 and 72.5%.

**Discussion:**

The overall intermethod agreement was moderate. This inconsistency could be explained by the diversity of assay methods, antigens used and immunoglobulin isotype tested. Estimated sensitivities were low, highlighting the limited value of antibody detection in CoVID-19.

**Conclusion:**

Comparison of five systems for SARS-CoV-2 IgG and IgM antibodies showed limited sensitivity and overall concordance. The place and indications of serological status assessment with currently available tools in the CoVID-19 pandemic need further evaluations.

## Introduction

A new Coronavirus pandemic has emerged in December 2019, in Wuhan, China. In 7 months, more than nine million people were infected by Severe Acute Respiratory Syndrome – Coronavirus 2 (SARS-CoV-2), so called because of high sequence homology with SARS-CoV (Li X. et al., 2020). This Coronavirus Disease 2019 (or CoVID-19) displays a benign course in most subjects, but may cause pneumonia, acute respiratory distress syndrome (ARDS) and death in an estimated 5–10% of patients (Zhang et al., 2020; Zhou et al., 2020). Currently, the gold standard for CoVID-19 diagnosis is the SARS-CoV-2 RT-PCR, despite new molecular methods relying on LAMP (Park et al., 2020) or CRISPR-Cas9 technologies (Joung et al., 2020). Antigenic tests of viral proteins are cheaper and faster than molecular diagnosis but their sensitivity is low (Scohy et al., 2020). The determination of serological status may trace previous contact with SARS-CoV-2, and is instrumental for retrospective diagnosis or seroprevalence and epidemiological studies. In this work, we compared an in-house system and four commercial solutions relying on three methods, in order to determine specific advantages and pitfalls of each one of the five assays.

## Materials and Equipment

### In-House Indirect Immunofluorescence Assay

We developed an in-house indirect immunofluorescence assay (IFA) to detect anti-SARS-CoV-2 antibodies (Edouard et al., 2020). Briefly, Vero E6 cells infected with SARS-CoV-2 strain IHU-MI2 (full genome sequence of this strain available at the European Molecular Biology Laboratory, EMBL project accession no. PRJEB38023) were harvested between 24 and 48 h post-inoculation, washed and inactivated using 5% paraformaldehyde. Each well of a microscope glass slide was spotted with 50 nL of this solution (as antigen), uninfected cells (as negative control) and a clinical isolate of *Staphylococcus aureus* (as positive control for serum deposit) (Gouriet et al., 2008). Then, serum samples incubated 30 min at 56°C for complement inactivation, diluted from 1:25 to 1:1,600 for IgM determination and from 1:50 to 1:3,200 for IgG determination and pipetted onto slides. After a 30-minute incubation at 37°C and three washes with PBS, anti-IgG and anti-IgM conjugates (bioRad France, Marne-la-Coquette, France) were added, followed by a 30-minute incubation at 37°C (Table 1). There were no blocking steps. After washing, slides were observed under a fluorescence microscope (AxioSkop 40, Zeiss, Marly–le-Roi, France) by two independent operators. In case of discrepancy, a third operator read the well. For each serum, the presence or absence of anti-SARS-CoV-2 antibodies was reported only if technical validation of the corresponding spot had been successfully passed (absence of fluorescence in uninfected cells (negative control) and visible fluorescence in the *S. aureus* spot (positive control). IFA produced quantitative results through sequential titration.

**TABLE 1:** Comparison of the methods.

	**In-house immuno-fluorescent assay**	**EUROIMMUN ELISA SARS-CoV-2 IgG**	**NovaLisa SARS-CoV-2 IgG and IgM**	**T-Tek SARS-CoV-2 IgG/IgM antibody Test Kit**	**Sure Bio-tech SARS-CoV-2 IgM/IgG antibody Rapid Test**
Method	Indirect immunofluorescence	ELISA	ELISA	Immunochromatography	Immunochromatography
Class of antibodies	IgG, IgM, and IgA	IgG	IgG and IgM (2 kits)	IgG and IgM (1 cassette)	IgG and IgM (1 cassette)
Antigen(s)	Inactivated infected cells	Recombinant S1 protein	Recombinant N protein	Recombinant N, S, and RBD proteins	Recombinant N and S proteins
Time	±3 h	±2.5 h	±2 h	15 min	15 min
Medium	Serum or plasma	Serum or plasma	Serum or plasma	Serum, plasma, or whole blood	Serum, plasma, or whole blood
Test sample	25 μL	10 μL	10 μL	10 μL	10 μL
Interpretation	Fluorescence microscope reading	Determination of a score to cut-off ratio	Determination of a score to cut-off ratio	Direct reading	Direct reading

*N, nucleocapside protein; S, spike protein; RBD, receptor-binding domain.*

### ELISA

We evaluated two commercial ELISA kits: EUROIMMUN^®^ ELISA SARS-CoV-2 IgG (Euroimmun France^®^, Bussy Saint-Martin, France, hereafter called “Euroimmun”) and NovaLisa^®^ SARS-CoV-2 IgG and IgM (NovaTec^®^, Dietzenbach, Germany, hereafter called “NovaLisa”). Briefly, both were direct ELISA methods, using horseradish peroxidase (HRP)-labeled conjugate and tetramethylbenzidine (TMB) as revealing agent (Table 1). Assays were performed following manufacturers’ instructions, including duplicate testing. For each sample, the ratio between the mean optical density (OD) and the cut-off was calculated. For Euroimmun, antibodies were considered undetectable (negative result) if the ratio was lower than 0.8, inconclusive between 0.8 and 1.1 and positive above 1.1. For NovaLisa, ratios were expressed in NTU (NovaTec Unit) and cut-offs were 9 NTU, 9–11 NTU and higher than 11 NTU for, respectively, negative, inconclusive, and positive interpretation. For each ELISA kit, negative and positive controls were assessed and successfully passed.

### Lateral Flow Assays

We evaluated two lateral flow assays (LFA) systems: T-Tek^®^ SARS-CoV-2 IgG/IgM Antibody Test Kit (T-Tek^®^, Villefranche-sur-Saône, France, hereafter called “T-Tek”) and Sure Bio-tech^®^ SARS-CoV-2 IgM/IgG Antibody Rapid Test (New York, NY, United States, hereafter called “Sure Bio-tech”). Briefly, these cassettes are immunochromatographic assays, using a capture method for qualitative detection of SARS-CoV-2 IgM and IgG antibodies (Table 1). Direct observation was performed by two independent operators and a qualitative result (positive or negative) was reported. No discrepancy between operator for LFA reading has been reported.

## Patients and Methods

### Patients and Samples

Adult patients (*n* = 40) with RT-PCR-confirmed SARS-CoV-2 infection (Amrane et al., 2020) attending the Méditerranée Infection University Hospital Institute (Assistance Publique—Hôpitaux de Marseille, France) were assessed for SARS-CoV-2 antibodies as part of their routine medical follow-up. Sera were collected and immediately frozen at −80°C. Demographic characteristics, risk factors, disease severity (National Institutes Health, 2020), laboratory, and outcome data were extracted from electronic medical records, retrospectively analyzed and are presented in Table 2. We also selected 10 sera which had been collected in 2019 from patients free of any Coronavirus infection.

**TABLE 2:** Demographic and laboratory findings of the study cohort.

***n* = 40**	
Age (y.o.; med ± 5–95 percentile)	39.9 (23.6–63.8)
Sex-ratio M/F	23/17
Symptomatic at diagnosis	92,5 (37/40)
Virus load at diagnosis (Ct; med ± 5–95 percentile)	25.9 (20.0–34.9)
Risk factors [*n* (%)]	16/40 (40%)
>70 y.o.	1 (2.5%)
CV disease	3 (7.5%)
Active smokers	6 (15.0%)
Diabetes	3 (7.5%)
Chronic lung disease	2 (5.0%)
End-stage renal disease	0
Cancer	0
Secondary ID	3 (7.5%)
Cirrhosis	0
BMI > 40	0
Outcome [*n* (%)]	
Hospitalized	4 (10.0%)
ARDS	2 (5.0%)
ICU	1 (2.5%)
Death	0

*ARDS, acute respiratory distress syndrome; BMI, body Mass Index; CV, cardiovascular; Ct, cycle threshold; ICU, intensive Care Unit; ID, immunodepression; y.o., years old.*

### Data Analysis

Immunofluorescence assay, ELISA, and LFA results were expressed as positive or negative. Inconclusive ELISA results were considered as negative for statistical analysis. Significant associations between variables were searched using chi-square test (or Fisher’s exact test to prevent overestimation of statistical significance for small data sets) and determination of agreement rate and Cohen’s Kappa. The significance threshold was set at *p* < 0.05.

We studied the serological response according to the time of serum sampling related to the reported date of COVID-19 symptom onset. Estimated sensitivity of the tests was calculated based on the assumption that specific IgM and IgG should be detectable 10 days after the onset of disease. Indeed, previous studies on antibody kinetics demonstrated that detection of SARS-CoV-2 antibodies before 10 days was uncommon (Guo et al., 2020; Okba et al., 2020).

## Results

### Demographic Findings

**FIGURE 1:**
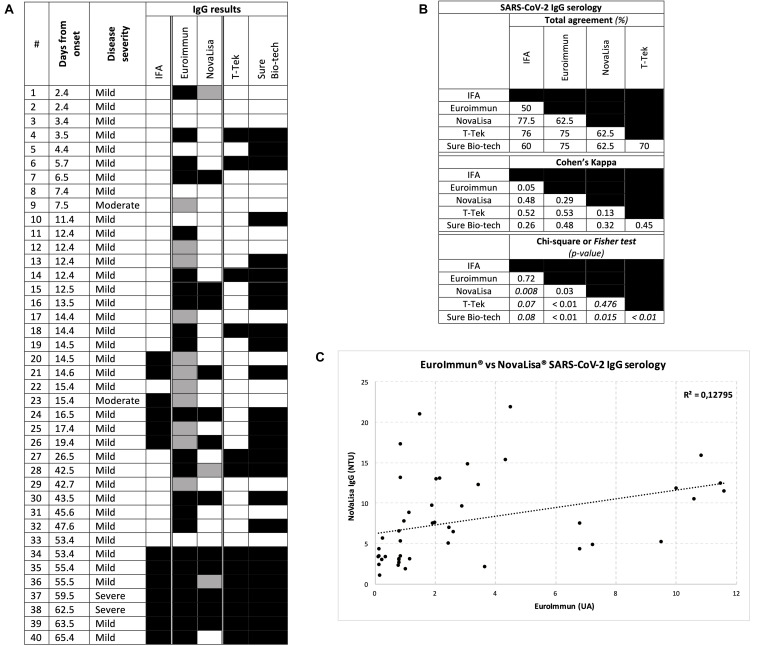
Results for IgG serology. **(A)** Individual CoVID-19 patient results. Each row represents a patient, each column a serological test, a black block a positive result, a gray block an inconclusive result (for ELISA methods), and a white block a negative result **(B)** Total agreement, Cohen’s Kappa value and Chi-square (or Fisher’s exact test in italic) *p*-value between two tests **(C)** Correlation plot between the two ELISA methods.

**FIGURE 2:**
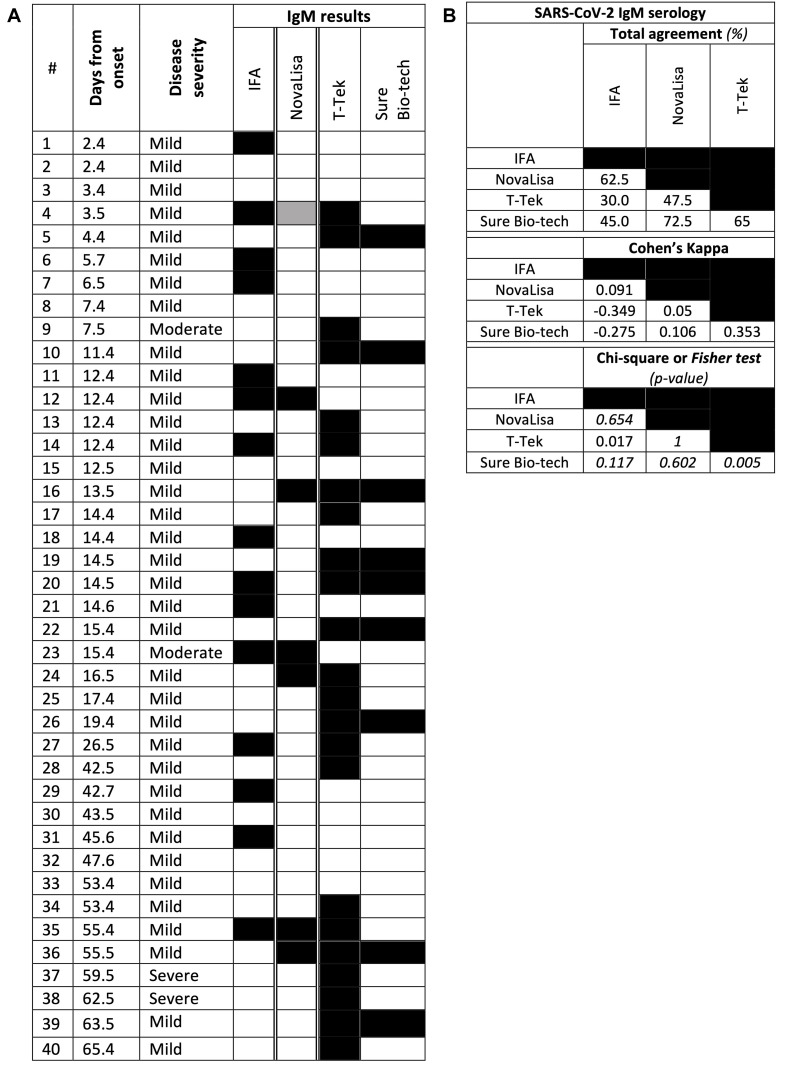
Results for IgM serology. **(A)** Individual CoVID-19 patient results. Each row represents a patient, each column a serological test, a black block a positive result, a gray block an inconclusive result (for ELISA methods), and a white block a negative result **(B)** Total agreement, Cohen’s Kappa value and Chi-square (or Fisher’s exact test in italic) *p*-value between two tests.

The median age of patients was 39.9 years (5–95 percentile: 23.6–63.8). A majority were men (57.5%) and had mild or moderate clinical presentation with 10% patients requiring hospital admission, two patients (patients #37 and #38) being diagnosed with Acute Respiratory Distress Syndrome (ARDS, 5%), and no fatality. Sixteen patients (40%) had one risk factor of severe disease (hypertension, obesity, or diabetes mellitus), and only one had two such underlying conditions. The median virus load at diagnosis was measured at 25.9 Ct (i.e., 480,950 genome copies/mL), range 20.0–34.9 (941–25 million genome copies/mL). For the two patients with ARDS, samples were collected 60 days after disease onset. For the two other patients requiring hospitalization, samples were collected 8 (patient #9) and 15 (patient #23) days after onset. Individual results of IgG and IgM assays are shown in Figures 1A, 2A, respectively.

### Detection of SARS-CoV-2 IgG

Anti-SARS-CoV-2 IgG detection was included in the five assays (Figure 1). Control sera were anti-SARS-CoV-2 IgG negative with all assays (data not shown). In patients, Intermethod comparison for IgG determination was 50% or higher (Figure 1B). In-house IFA and EuroImmun had moderate agreement with all other systems (at least 60% agreement and 0.26 Cohen’s Kappa), except with each other (Cohen’s Kappa 0.05 and Chi-square *p*-value at 0.72). T-Tek and NovaLisa had also moderate agreement with all the systems (at least 62.5% agreement and 0.29 Cohen’s Kappa), except with each other (Cohen’s Kappa 0.13, Fisher test *p*-value at 0.476). Sure Bio-tech displayed a fair agreement with all the systems (agreement 60–75%; Cohen’s Kappa 0.26–0.48; Chi-square or Fisher test all significant). The correlation plot between the two ELISA systems (Figure 1C) revealed a significant correlation between EuroImmun and NovaLisa IgG results (correlation coefficient = 0.35; *p*-value of Pearson’s test = 0.025), despite the fact that most positive results with EuroImmun were weak (between 1.1 and 4), which may explain the modest correlation coefficient. Best estimated sensitivities (Figure 1A and Table 3) were found with Sure Bio-tech and EuroImmun (respectively, 71.0 and 61.3%), whereas the sensitivity of other methods was less than 50%. Regarding the two patients with ARDS, IgG were detected with all the systems. For the two other hospitalized patients, there was only an inconclusive result for patients #9 and #23 with EuroImmun, and a positive result for #23 with IFA.

**TABLE 3:** Estimated sensitivities for IgG and IgM results.

	**Sensitivity for IgG serology**	**Sensitivity for IgM serology**	**Sensitivity for IgG + IgM serology**
IFA	41.9% (13/31)	35.5% (11/31)	64.5% (20/31)
EuroImmun	61.3% (19/31)		
NovaLisa	35.5% (11/31)	19.4% (6/31)	45.2% (14/31)
T-Tek	35.5% (11/31)	64.5% (20/31)	67.7% (21/31)
Sure Bio-tech	71.0% (22/31)	25.9% (8/31)	80.7% (25/31)

### Detection of SARS-CoV-2 IgM

Anti-SARS-CoV-2 IgM detection was available with four evaluated assays (Figure 2). Control sera were anti-SARS-CoV-2 IgM negative with all assays (data not shown). The best intermethod agreement was between the two LFA solutions Sure Bio-tech and T-Tek (65% 0.353 of Cohen’s Kappa and Fisher test *p*-value at 0.005), otherwise ranging from 30 to 72.5% agreement, −0.349–0.106 of Cohen’s Kappa. Despite a significant Chi-square test between T-Tek and IFA (*p* = 0.017), agreement was low (negative Cohen’s Kappa and 30% of agreement). Estimated sensitivities (Figure 2A and Table 3) were 64.5% for T-Tek but only 19.4–35.5% for IFA, NovaLisa and Sure Bio-tech. In the two ARDS patients, anti-SARS-CoV-2 IgM were detected only with T-Tek. For the two other hospitalized patients, #9 had a positive result with T-Tek only whereas #23 had a positive result with IFA and NovaLisa.

## Discussion

This study addressed the analytical performance of five serological assays for SARS-CoV-2, by means of a panel of in-house and commercial, qualitative and quantitative, manual and automated methods and solutions. One in-house IFA method, two commercial ELISA kits and two commercial LFA kits were applied to serum samples from 40 RT-PCR-confirmed SARS-CoV-2 infected patients and 10 coronavirus-negative controls collected before the SARS-CoV-2 outbreak.

There was a fair to moderate agreement between all the tests. Intermethod comparison revealed diverging results, stemming in the choices of assay method and antigen, which are the main challenges of SARS-CoV-2 serology. IFA is the most time-consuming method and requires a biosafety level 3 laboratory for the culture and handling of infected cells, but it uses whole viral antigen extracts, allowing antibody detection against virtually any viral protein. However, assays relying on recombinant proteins are more accurate and may be easier to standardize, due to higher reproducibility and similar immunoreactivity whereas “natural” proteins display more variability. ELISA methods are robust and can easily be automated as a load-and-go system, generating dozens of results in a couple of hours, but there is an inconclusive (“gray”) zone with undetermined results. Other studies have compared ELISA tests for CoVID-19, with good sensitivity and great overall intermethod agreement (Elslande et al., 2020; Nicol et al., 2020; Theel et al., 2020). Despite the need of quantitative assessment of positive results, LFA is a quick and easy-to-handle method, which does not require specialized training prior to its implementation (Li Z. et al., 2020). Different results may also be explained by variation in the antigens used in the assays (Table 1). NovaLisa method only targets recombinant nucleocapside (N) protein, despite the key role of spike (S) protein in the viral entrance, which is displayed all around the surface of the virus (Hoffmann et al., 2020). Moreover, the amino acid sequence of N protein, and especially the N-terminal domain, is highly conserved in all beta-coronaviruses and may cause false positive results and/or fail to detect true early sensitizations. Other methods target the S protein alone or in combination, as it may be one of the most immunogenic SARS-CoV-2 proteins (Lan et al., 2020; Ni et al., 2020). Other systems have been developed and are promising in this context, such as chemiluminescence immunoassays or microsphere-based suspension array technologies (Hou et al., 2020; Kohmer et al., 2020).

Poor estimated sensitivities of IgG and IgM determination were striking. To our knowledge, serum IgM may decrease rapidly, potentially accounting for apparently poor assay sensitivity. Previous studies reported slightly better sensitivity results, 66.7–98% for IgG and 60–95% for IgM (Beavis et al., 2020; Guo et al., 2020; Hou et al., 2020; Kohmer et al., 2020; Li Z. et al., 2020; Ma et al., 2020; Nagappa and Marimuthu, 2020; Qu et al., 2020; Traugott et al., 2020; Tuaillon et al., 2020; Van Elslande et al., 2020; Zhao et al., 2020). It is difficult to accurately determine the clinical performance without a gold standard method, therefore we calculated an “estimated” sensitivity: we assumed that antibodies were present at least 10 days after the onset of the symptoms. Very early production of antibodies is unusual in viral respiratory infections (Allie and Randall, 2017). Median time for first IgG detection was 14 days (IQR 10–18) after symptom onset (Guo et al., 2020). In the CoVID-19 context, detection of peripheral antibodies a few days after symptom onset or molecular diagnosis might be explained by a longer incubation period, a late diagnosis for asymptomatic patients or a false positive result. Otherwise, production of detectable antibody levels may require a longer time, as observed in previous Coronavirus outbreaks (Tang et al., 2004; Ko et al., 2017), and especially in immunocompromised patients.

It has been suggested that antibody-dependent mechanisms play a major role during immune responses against SARS-CoV-2, and may depend on the development of virus-specific CD4^+^ and CD8^+^ T cell immunity (St John and Rathore, 2020; Vabret et al., 2020). However, antibodies produced after SARS-CoV-2 infection inconsistently carry neutralizing activity (Seydoux et al., 2020). Taken together, current data suggest that SARS-CoV-2 serological assays may be useful as indirect biomarkers of prior contact with SARS-CoV-2 but not of individual protection against reinfection. In this study, we showed that the sensitivity of five serology solutions was comparable, albeit intermethod agreement were not optimal. Thorough analytical characterization and quality assessment should be performed by each laboratory once a method is chosen for routine investigation in patients.

## Conclusion

Comparison of five commercial and in-house assays for anti-SARS-CoV-2 IgG and IgM antibodies found limited sensitivity and overall concordance. Whether this result was due to coated antigens, analytical processes or anti-SARS-CoV-2 antibody kinetics and magnitude in patients warrants further investigations. The place and indications of serological status assessment with currently available tools in the CoVID-19 pandemic need reevaluation.

## Data Availability Statement

The original contributions presented in the study are included in the article/supplementary material, further inquiries can be directed to the corresponding author/s.

## Ethics Statement

Ethical review and approval was not required for the study on human participants in accordance with the local legislation and institutional requirements. Written informed consent for participation was not required for this study in accordance with the national legislation and the institutional requirements.

## Author Contributions

MM, AB, MD, and JV contributed to the conception and design of the study. MM and AB performed the experiments. SE, FF, and FD performed the statistical analysis. J-LM wrote the first draft of the manuscript. All authors contributed to manuscript revision, read and approved the submitted version.

## Conflict of Interest

The two ELISA kits and lateral flow assays were kindly provided by suppliers for evaluation. The authors declare that the research was conducted in the absence of any commercial or financial relationships that could be construed as a potential conflict of interest.
